# 3-(4-Chloro­phen­oxy)-1-(4-meth­oxy­phen­yl)-4-(4-nitro­phen­yl)azetidin-2-one

**DOI:** 10.1107/S1600536811013018

**Published:** 2011-04-13

**Authors:** Ray J. Butcher, Mehmet Akkurt, Aliasghar Jarrahpour, Seid Ali Torabi Badrabady

**Affiliations:** a Department of Chemistry, Howard University, 525 College Street NW, Washington, DC 20059, USA; b Department of Physics, Faculty of Sciences, Erciyes University, 38039 Kayseri, Turkey; c Department of Chemistry, College of Sciences, Shiraz University, 71454 Shiraz, Iran

## Abstract

In the title compound, C_22_H_17_ClN_2_O_5_, the nearly planar four-membered β-lactam ring [maximum deviation of 0.016 (1) for the N atom] makes dihedral angles of 53.07 (9), 73.19 (9) and 6.61 (9)° with the chloro-, nitro- and meth­oxy­benzene rings, respectively. The crystal structure is stabilized by C—H⋯O hydrogen bonds, a weak C—H⋯π inter­action and a π–π stacking inter­action [centroid–centroid distance = 3.6513 (8) Å] between the meth­oxy­benzene rings of inversion-related mol­ecules.

## Related literature

For general background to β-lactams, see: Banik *et al.* (2004 ▶); Garud *et al.* (2009 ▶); Jarrahpor & Khalili (2007 ▶); Jarrahpour & Zarei (2006 ▶, 2010 ▶). For some of our previous reports of the structures of β-lactams, see: Akkurt *et al.* (2008*a* ▶,*b* ▶, 2011*a* ▶,*b* ▶); Baktır *et al.* (2009 ▶); Yalçın *et al.* (2009 ▶); Çelik *et al.* (2009 ▶).
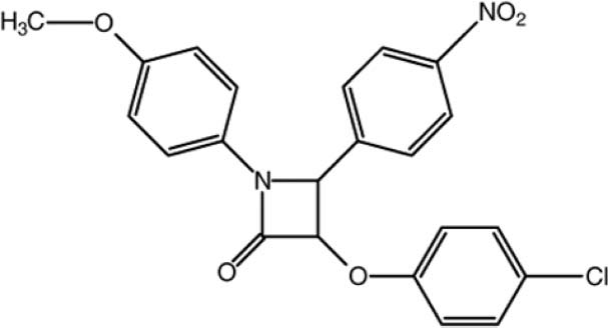


## Experimental

### 

#### Crystal data

C_22_H_17_ClN_2_O_5_
*M* *_r_* = 424.83
Monoclinic, 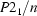

*a* = 6.0863 (2) Å
*b* = 20.0855 (7) Å
*c* = 17.3819 (7) Å
β = 97.419 (4)°
*V* = 2107.09 (13) Å^3^
*Z* = 4
Mo *K*α radiation
μ = 0.22 mm^−1^
*T* = 123 K
0.49 × 0.17 × 0.14 mm

#### Data collection

Oxford Diffraction Xcalibur Ruby Gemini diffractometer
Absorption correction: multi-scan (*CrysAlis PRO*; Oxford Diffraction, 2007 ▶) *T*_min_ = 0.901, *T*_max_ = 0.970
20727 measured reflections
10522 independent reflections
7301 reflections with *I* > 2σ(*I*)
*R*_int_ = 0.041

#### Refinement

*R*[*F* ^2^ > 2σ(*F* ^2^)] = 0.074
*wR*(*F* ^2^) = 0.190
*S* = 1.08
10522 reflections
272 parameters
H-atom parameters constrained
Δρ_max_ = 0.67 e Å^−3^
Δρ_min_ = −0.58 e Å^−3^

### 

Data collection: *CrysAlis PRO* (Oxford Diffraction, 2007 ▶); cell refinement: *CrysAlis PRO*; data reduction: *CrysAlis RED* (Oxford Diffraction, 2007 ▶); program(s) used to solve structure: *SHELXS97* (Sheldrick, 2008 ▶); program(s) used to refine structure: *SHELXL97* (Sheldrick, 2008 ▶); molecular graphics: *ORTEP-3 for Windows* (Farrugia, 1997 ▶); software used to prepare material for publication: *WinGX* (Farrugia, 1999 ▶) and *PLATON* (Spek, 2009 ▶).

## Supplementary Material

Crystal structure: contains datablocks global, I. DOI: 10.1107/S1600536811013018/su2266sup1.cif

Structure factors: contains datablocks I. DOI: 10.1107/S1600536811013018/su2266Isup2.hkl

Additional supplementary materials:  crystallographic information; 3D view; checkCIF report

## Figures and Tables

**Table 1:** Hydrogen-bond geometry (Å, °) *Cg*4 is the centroid of the C16–C21 benzene ring.

*D*—H⋯*A*	*D*—H	H⋯*A*	*D*⋯*A*	*D*—H⋯*A*
C2—H2*A*⋯O4^i^	1.00	2.45	3.287 (2)	141
C3—H3*A*⋯O1^ii^	1.00	2.29	3.2439 (17)	159
C20—H20*A*⋯O1^iii^	0.95	2.50	3.3086 (18)	144
C21—H21*A*⋯O1	0.95	2.52	3.1397 (18)	123
C6—H6*A*⋯*Cg*4^iv^	0.95	2.71	3.4145 (17)	131

Symmetry codes: (i) 
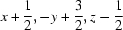
; (ii) 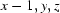; (iii) 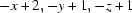; (iv) 
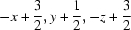
.

**Table 2:** The dihedral angles between the mean planes of the rings in the title mol­ecule (°)

	Ring 2	Ring 3	Ring 4
Ring 1	53.07 (9)	73.19 (9)	6.61 (9)
Ring 2		64.42 (7)	46.85 (7)
Ring 3			79.45 (7)

Ring 1 is the N1/C1–C3 β-lactam ring, ring 2 is the C4–C9 benzene ring, ring 3 is the C10–C15 benzene ring and ring 4 is C16–C21 benzene ring.

**Fig. 1.**
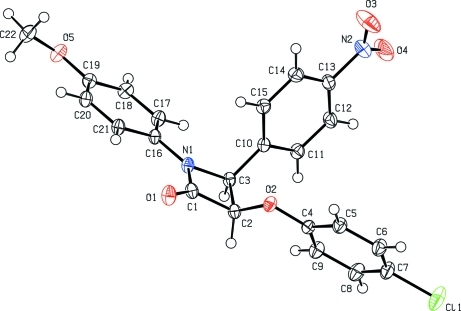
Molecular structure of the title compound showing the atom labeling scheme. Displacement ellipsoids for non-H atoms are drawn at the 50% probability level.

**Fig. 2.**
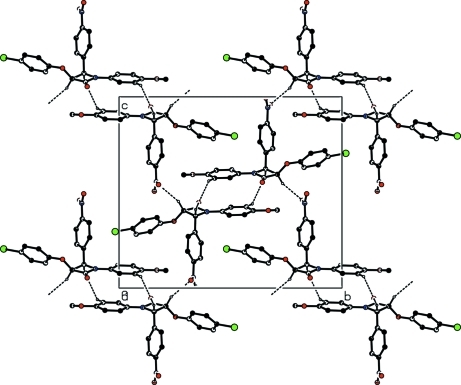
The crystal packing and C-H···O hydrogen-bond interactions (dashed lines) of the title compound viewed down the a axis.

## supplementary crystallographic information

### Comment

The β-lactam antibiotics consists of a strained, four-membered, heterocyclic ring, known as the β-lactam ring (Garud *et al.*, 2009). The most literal definition of a β-lactam antibiotics are the monocyclic β-lactams that do not contain another ring fused to the β-lactam one (Jarrahpour & Zarei, 2006). The discovery of the monocyclic β-lactams suggesting that the biological activity of β-lactams was strictly correlated to the presence of a suitably functionalized β-lactam ring (Jarrahpour & Zarei, 2010; Banik *et al.*, 2004). The β-lactam ring systems show many interesting biological properties, such as cholesterol absorption inhibitors, human cytomegalovirus (HCMV) protease inhibitors, thrombin inhibitors, antihyperglycemic, anti-tumour, anti-HIV, human leukocyte elastase (HLE), potential antimalarials, anti-influenza virus, and serine-dependent enzyme inhibitors (Jarrahpor & Khalili, 2007).

As an extension of our work (Baktır *et al.*, 2009; Çelik *et al.*, 2009; Yalçın *et al.*, 2009; Akkurt *et al.*, 2008*a*,**b*;* Akkurt *et al.*, 2011*a*,*b*) on structural characterization of the β-lactam compounds, we herein report on the X-ray crystal structure of the title compound.

In the title molecule, Fig. 1, the β-lactam ring (N1/C1–C3) is nearly planar, with maximum deviations of -0.016 (1) for N1 and 0.015 (1) Å for C1. The C1—N1—C16—C17, N1–C3—C10—C11, O1—C1—C2—O2, C3—C2—O2—C4 and C2—O2—C4—C5 torsion angles are -172.79 (14), -159.77 (12), -60.7 (2), 92.79 (14) and -168.40 (12) °, respectively. The dihedral angles between the ring planes are listed in Table 2.

In the crystal molecules are linked by intermolecular C—H···O hydrogen-bond interactions and a weak C—H···π interaction (Table 1 and Fig. 2). Furthermore, there is a π-π stacking interaction [*Cg*4···*Cg*4^i^ = 3.6513 (8) Å, where *Cg*4 is a centroid of the C16–C21 benzene ring; symmetry code: (i) = 1 - *x*, 1 - *y*, 1 - *z*] between the benzene rings attached to the methoxy group of molecules related by an inversion center.

### Experimental

A solution of *N*-(4-nitrobenzylidene)-4-methoxybenzenamine (1.00 mmol) was stirred with 4-chlorophenoxy acetic acid (1.50 mmol), *p*-toluenesulfonyl chloride (1.50 mmol) and triethylamine (2.5 mmol) in dry CH_2_Cl_2_ at room temperature over night. Then it was washed with HCl 1 N (20 ml), saturated NaHCO_3_ (20 ml), brine (20 ml), dried over Na_2_SO_4_ and the solvent was evaporated under reduced pressure to give the crude product which was then purified by column chromatography over silica gel (7:3 hexane-EtOAc). (Yield 78%; mp: 415–417 K). Elemental analysis: Calc. for C_22_H_17_ClN_2_O_5_: C, 62.20; H, 4.03; N, 6.59%; Found: C, 62.15; H, 4.07; N, 6.65%.

### Refinement

All H atoms were placed in their calculated positions and refined using a riding model: C—H = 0.98, 1.00 and 0.95 Å, for methyl, methine, and aromatic H-atoms, respectively, with U_iso_(H) = k × U_eq_(C), where k = 1.5 for the methyl H-atoms and 1.2 for all other H-atoms. In the crystal structure there is an 89 Å^3^ void, but the low electron density (0.67 e.Å^-3^) in the difference Fourier map suggests no solvent molecule occupying this void.

### Crystal data

**Table:**

C_22_H_17_ClN_2_O_5_	*F*(000) = 880
*M**_r_* = 424.83	*D*_x_ = 1.339 Mg m^−^^3^
Monoclinic, *P*2_1_/*n*	Mo *K*α radiation, λ = 0.71073 Å
Hall symbol: -P 2yn	Cell parameters from 6350 reflections
*a* = 6.0863 (2) Å	θ = 5.1–37.5°
*b* = 20.0855 (7) Å	µ = 0.22 mm^−^^1^
*c* = 17.3819 (7) Å	*T* = 123 K
β = 97.419 (4)°	Needle, colourless
*V* = 2107.09 (13) Å^3^	0.49 × 0.17 × 0.14 mm
*Z* = 4	

### Data collection

**Table:**

Oxford Diffraction Xcalibur Ruby Gemini diffractometer	10522 independent reflections
Radiation source: Enhance (Mo) X-ray Source	7301 reflections with *I* > 2σ(*I*)
graphite	*R*_int_ = 0.041
Detector resolution: 10.5081 pixels mm^-1^	θ_max_ = 37.6°, θ_min_ = 5.2°
ω scans	*h* = −10→6
Absorption correction: multi-scan (*CrysAlis PRO*; Oxford Diffraction, 2007)	*k* = −34→29
*T*_min_ = 0.901, *T*_max_ = 0.970	*l* = −29→27
20727 measured reflections	

### Refinement

**Table:**

Refinement on *F*^2^	Primary atom site location: structure-invariant direct methods
Least-squares matrix: full	Secondary atom site location: difference Fourier map
*R*[*F*^2^ > 2σ(*F*^2^)] = 0.074	Hydrogen site location: inferred from neighbouring sites
*wR*(*F*^2^) = 0.190	H-atom parameters constrained
*S* = 1.08	*w* = 1/[σ^2^(*F*_o_^2^) + (0.0816*P*)^2^ + 0.3529*P*] where *P* = (*F*_o_^2^ + 2*F*_c_^2^)/3
10522 reflections	(Δ/σ)_max_ = 0.001
272 parameters	Δρ_max_ = 0.67 e Å^−^^3^
0 restraints	Δρ_min_ = −0.58 e Å^−^^3^

### Special details

**Table:**

Experimental. Spectroscopic data for the title c ompound: IR (KBr, cm^-1^): 1744.5 (CO, β-lactam). ^1^H-NMR (250 MHz, CDCl_3_) δ (p.p.m.): 3.67 (OMe, s, 3H), 5.88 (H-4, d, 1H, *J* = 5.0), 5.95 (H-3, d, 1H, *J* = 5.0), 6.78–8.14 (aromatic protons as a doublet at 6.80, a doublet at 6.90, a doublet at 7.15, a doublet at 7.23, a doublet at 7.61, a doublet at 8.12, 12H). ^13^C-NMR (62.9 MHz, CDCl_3_) δ (p.p.m.): 55.6 (OMe), 60.3 (C-4), 81.3 (C-3), 115.1–156.6 (aromatic carbons), 161.8 (CO, β-lactam).
Geometry. Bond distances, angles *etc*. have been calculated using the rounded fractional coordinates. All su's are estimated from the variances of the (full) variance-covariance matrix. The cell e.s.d.'s are taken into account in the estimation of distances, angles and torsion angles
Refinement. Refinement on *F*^2^ for ALL reflections except those flagged by the user for potential systematic errors. Weighted *R*-factors *wR* and all goodnesses of fit *S* are based on *F*^2^, conventional *R*-factors *R* are based on *F*, with *F* set to zero for negative *F*^2^. The observed criterion of *F*^2^ > σ(*F*^2^) is used only for calculating -*R*-factor-obs *etc*. and is not relevant to the choice of reflections for refinement. *R*-factors based on *F*^2^ are statistically about twice as large as those based on *F*, and *R*-factors based on ALL data will be even larger.

### Fractional atomic coordinates and isotropic or equivalent isotropic displacement parameters (Å^2^)

**Table:**

	*x*	*y*	*z*	*U*_iso_*/*U*_eq_	
Cl1	0.37871 (8)	1.01738 (2)	0.70470 (3)	0.0430 (1)	
O1	0.95577 (17)	0.64289 (6)	0.55371 (7)	0.0251 (3)	
O2	0.74828 (16)	0.75343 (5)	0.65041 (6)	0.0193 (2)	
O3	0.5101 (3)	0.65839 (9)	0.99331 (8)	0.0549 (6)	
O4	0.1603 (3)	0.67723 (9)	0.95892 (8)	0.0507 (5)	
O5	0.54345 (18)	0.33051 (5)	0.59384 (7)	0.0258 (3)	
N1	0.62898 (18)	0.60647 (6)	0.60078 (7)	0.0175 (3)	
N2	0.3499 (3)	0.66717 (8)	0.94411 (8)	0.0341 (4)	
C1	0.7794 (2)	0.65032 (7)	0.57845 (8)	0.0184 (3)	
C2	0.6374 (2)	0.71011 (7)	0.59484 (8)	0.0176 (3)	
C3	0.4744 (2)	0.65738 (7)	0.62320 (8)	0.0170 (3)	
C4	0.6499 (2)	0.81410 (7)	0.66070 (8)	0.0175 (3)	
C5	0.7820 (2)	0.86043 (7)	0.70490 (8)	0.0222 (3)	
C6	0.6991 (3)	0.92302 (8)	0.71847 (9)	0.0258 (4)	
C7	0.4823 (3)	0.93834 (8)	0.68819 (10)	0.0266 (4)	
C8	0.3492 (2)	0.89266 (8)	0.64457 (10)	0.0263 (4)	
C9	0.4332 (2)	0.82983 (7)	0.63096 (9)	0.0224 (3)	
C10	0.4462 (2)	0.65906 (7)	0.70792 (7)	0.0165 (3)	
C11	0.2574 (2)	0.68941 (7)	0.72993 (8)	0.0202 (3)	
C12	0.2254 (2)	0.69232 (8)	0.80772 (8)	0.0229 (4)	
C13	0.3852 (3)	0.66479 (7)	0.86185 (8)	0.0223 (3)	
C14	0.5750 (3)	0.63433 (8)	0.84242 (8)	0.0232 (3)	
C15	0.6038 (2)	0.63173 (7)	0.76442 (8)	0.0202 (3)	
C16	0.6116 (2)	0.53648 (7)	0.59893 (7)	0.0166 (3)	
C17	0.4208 (2)	0.50585 (7)	0.61868 (8)	0.0196 (3)	
C18	0.4028 (2)	0.43687 (7)	0.61633 (8)	0.0207 (3)	
C19	0.5751 (2)	0.39828 (7)	0.59468 (8)	0.0193 (3)	
C20	0.7656 (2)	0.42899 (7)	0.57502 (8)	0.0203 (3)	
C21	0.7837 (2)	0.49782 (7)	0.57686 (8)	0.0194 (3)	
C22	0.7363 (3)	0.29027 (8)	0.59211 (10)	0.0280 (4)	
H2A	0.57270	0.73370	0.54650	0.0210*	
H3A	0.32890	0.65630	0.58920	0.0200*	
H5A	0.92920	0.84910	0.72580	0.0270*	
H6A	0.78900	0.95500	0.74800	0.0310*	
H8A	0.20170	0.90400	0.62410	0.0320*	
H9A	0.34280	0.79790	0.60150	0.0270*	
H11A	0.15010	0.70820	0.69150	0.0240*	
H12A	0.09710	0.71270	0.82310	0.0270*	
H14A	0.68190	0.61580	0.88120	0.0280*	
H15A	0.73230	0.61110	0.74950	0.0240*	
H17A	0.30350	0.53210	0.63370	0.0230*	
H18A	0.27280	0.41600	0.62950	0.0250*	
H20A	0.88320	0.40270	0.56030	0.0240*	
H21A	0.91320	0.51870	0.56310	0.0230*	
H22A	0.69550	0.24320	0.59390	0.0420*	
H22B	0.84530	0.30090	0.63700	0.0420*	
H22C	0.80030	0.29910	0.54420	0.0420*	

### Atomic displacement parameters (Å^2^)

**Table:**

	*U*^11^	*U*^22^	*U*^33^	*U*^12^	*U*^13^	*U*^23^
Cl1	0.0412 (2)	0.0256 (2)	0.0610 (3)	0.0126 (2)	0.0016 (2)	−0.0114 (2)
O1	0.0172 (4)	0.0272 (5)	0.0331 (5)	−0.0022 (4)	0.0115 (4)	−0.0070 (4)
O2	0.0166 (4)	0.0173 (4)	0.0233 (4)	0.0020 (3)	0.0005 (3)	−0.0029 (3)
O3	0.0564 (9)	0.0853 (13)	0.0211 (6)	−0.0004 (9)	−0.0024 (6)	−0.0015 (7)
O4	0.0568 (9)	0.0691 (11)	0.0309 (7)	0.0223 (8)	0.0234 (6)	0.0011 (6)
O5	0.0219 (5)	0.0174 (5)	0.0383 (6)	0.0011 (4)	0.0049 (4)	−0.0006 (4)
N1	0.0132 (4)	0.0185 (5)	0.0218 (5)	0.0004 (4)	0.0063 (4)	−0.0034 (4)
N2	0.0454 (8)	0.0378 (8)	0.0200 (6)	0.0035 (7)	0.0076 (6)	−0.0022 (5)
C1	0.0154 (5)	0.0212 (6)	0.0191 (5)	−0.0009 (4)	0.0039 (4)	−0.0037 (4)
C2	0.0159 (5)	0.0188 (5)	0.0184 (5)	0.0001 (4)	0.0029 (4)	−0.0021 (4)
C3	0.0125 (4)	0.0196 (6)	0.0190 (5)	0.0020 (4)	0.0027 (4)	−0.0026 (4)
C4	0.0168 (5)	0.0166 (5)	0.0193 (5)	0.0010 (4)	0.0027 (4)	−0.0002 (4)
C5	0.0217 (6)	0.0207 (6)	0.0230 (6)	0.0017 (5)	−0.0015 (5)	−0.0030 (5)
C6	0.0273 (6)	0.0210 (6)	0.0277 (7)	0.0020 (6)	−0.0020 (5)	−0.0049 (5)
C7	0.0288 (7)	0.0198 (6)	0.0312 (7)	0.0055 (6)	0.0034 (6)	−0.0038 (5)
C8	0.0194 (6)	0.0236 (7)	0.0352 (8)	0.0047 (5)	0.0008 (5)	−0.0014 (6)
C9	0.0165 (5)	0.0206 (6)	0.0294 (7)	0.0008 (5)	0.0006 (5)	−0.0025 (5)
C10	0.0136 (4)	0.0170 (5)	0.0190 (5)	−0.0001 (4)	0.0030 (4)	−0.0021 (4)
C11	0.0163 (5)	0.0228 (6)	0.0221 (6)	0.0035 (5)	0.0045 (4)	−0.0009 (5)
C12	0.0224 (6)	0.0261 (7)	0.0216 (6)	0.0028 (5)	0.0080 (5)	−0.0027 (5)
C13	0.0277 (6)	0.0219 (6)	0.0180 (5)	−0.0021 (5)	0.0059 (5)	−0.0033 (5)
C14	0.0245 (6)	0.0235 (6)	0.0208 (6)	0.0003 (5)	−0.0002 (5)	0.0006 (5)
C15	0.0168 (5)	0.0216 (6)	0.0219 (6)	0.0026 (5)	0.0016 (4)	−0.0008 (5)
C16	0.0139 (4)	0.0176 (5)	0.0185 (5)	0.0003 (4)	0.0028 (4)	−0.0032 (4)
C17	0.0134 (4)	0.0201 (6)	0.0257 (6)	0.0011 (4)	0.0046 (4)	−0.0020 (5)
C18	0.0147 (5)	0.0216 (6)	0.0260 (6)	−0.0010 (5)	0.0035 (4)	0.0006 (5)
C19	0.0178 (5)	0.0190 (6)	0.0208 (6)	−0.0002 (5)	0.0013 (4)	−0.0014 (4)
C20	0.0175 (5)	0.0210 (6)	0.0233 (6)	0.0027 (5)	0.0056 (4)	−0.0029 (5)
C21	0.0157 (5)	0.0204 (6)	0.0231 (6)	0.0004 (5)	0.0063 (4)	−0.0028 (5)
C22	0.0270 (7)	0.0204 (6)	0.0372 (8)	0.0041 (6)	0.0069 (6)	0.0013 (6)

### Geometric parameters (Å, °)

**Table:**

Cl1—C7	1.7456 (17)	C13—C14	1.387 (2)
O1—C1	1.2160 (16)	C14—C15	1.390 (2)
O2—C2	1.4061 (17)	C16—C17	1.3957 (18)
O2—C4	1.3795 (17)	C16—C21	1.3968 (18)
O3—N2	1.224 (2)	C17—C18	1.390 (2)
O4—N2	1.231 (3)	C18—C19	1.3943 (18)
O5—C19	1.3746 (17)	C19—C20	1.3942 (18)
O5—C22	1.429 (2)	C20—C21	1.387 (2)
N1—C1	1.3625 (18)	C2—H2A	1.0000
N1—C3	1.4756 (18)	C3—H3A	1.0000
N1—C16	1.4098 (19)	C5—H5A	0.9500
N2—C13	1.474 (2)	C6—H6A	0.9500
C1—C2	1.5277 (19)	C8—H8A	0.9500
C2—C3	1.5733 (19)	C9—H9A	0.9500
C3—C10	1.5048 (18)	C11—H11A	0.9500
C4—C5	1.3940 (19)	C12—H12A	0.9500
C4—C9	1.3894 (18)	C14—H14A	0.9500
C5—C6	1.386 (2)	C15—H15A	0.9500
C6—C7	1.391 (3)	C17—H17A	0.9500
C7—C8	1.384 (2)	C18—H18A	0.9500
C8—C9	1.393 (2)	C20—H20A	0.9500
C10—C11	1.3971 (18)	C21—H21A	0.9500
C10—C15	1.3938 (18)	C22—H22A	0.9800
C11—C12	1.3920 (19)	C22—H22B	0.9800
C12—C13	1.379 (2)	C22—H22C	0.9800
			
C2—O2—C4	117.25 (10)	O5—C19—C18	116.37 (12)
C19—O5—C22	116.49 (11)	O5—C19—C20	123.73 (12)
C1—N1—C3	95.86 (11)	C18—C19—C20	119.90 (13)
C1—N1—C16	133.57 (12)	C19—C20—C21	120.10 (12)
C3—N1—C16	130.43 (11)	C16—C21—C20	120.02 (12)
O3—N2—O4	124.14 (15)	O2—C2—H2A	113.00
O3—N2—C13	118.06 (17)	C1—C2—H2A	113.00
O4—N2—C13	117.79 (15)	C3—C2—H2A	113.00
O1—C1—N1	132.67 (14)	N1—C3—H3A	112.00
O1—C1—C2	135.15 (13)	C2—C3—H3A	112.00
N1—C1—C2	92.17 (10)	C10—C3—H3A	112.00
O2—C2—C1	112.44 (10)	C4—C5—H5A	120.00
O2—C2—C3	117.87 (11)	C6—C5—H5A	120.00
C1—C2—C3	85.64 (10)	C5—C6—H6A	121.00
N1—C3—C2	86.24 (9)	C7—C6—H6A	120.00
N1—C3—C10	115.54 (11)	C7—C8—H8A	120.00
C2—C3—C10	116.60 (11)	C9—C8—H8A	120.00
O2—C4—C5	115.60 (11)	C4—C9—H9A	120.00
O2—C4—C9	124.03 (12)	C8—C9—H9A	120.00
C5—C4—C9	120.36 (13)	C10—C11—H11A	120.00
C4—C5—C6	120.17 (13)	C12—C11—H11A	120.00
C5—C6—C7	119.00 (15)	C11—C12—H12A	121.00
Cl1—C7—C6	118.97 (13)	C13—C12—H12A	121.00
Cl1—C7—C8	119.66 (13)	C13—C14—H14A	121.00
C6—C7—C8	121.37 (15)	C15—C14—H14A	121.00
C7—C8—C9	119.47 (13)	C10—C15—H15A	120.00
C4—C9—C8	119.62 (13)	C14—C15—H15A	120.00
C3—C10—C11	118.66 (11)	C16—C17—H17A	120.00
C3—C10—C15	121.71 (11)	C18—C17—H17A	120.00
C11—C10—C15	119.63 (12)	C17—C18—H18A	120.00
C10—C11—C12	120.50 (12)	C19—C18—H18A	120.00
C11—C12—C13	118.11 (13)	C19—C20—H20A	120.00
N2—C13—C12	118.08 (15)	C21—C20—H20A	120.00
N2—C13—C14	118.74 (14)	C16—C21—H21A	120.00
C12—C13—C14	123.17 (13)	C20—C21—H21A	120.00
C13—C14—C15	117.90 (14)	O5—C22—H22A	109.00
C10—C15—C14	120.69 (13)	O5—C22—H22B	109.00
N1—C16—C17	119.76 (12)	O5—C22—H22C	109.00
N1—C16—C21	120.26 (11)	H22A—C22—H22B	109.00
C17—C16—C21	119.99 (13)	H22A—C22—H22C	109.00
C16—C17—C18	119.81 (12)	H22B—C22—H22C	110.00
C17—C18—C19	120.19 (12)		
			
C4—O2—C2—C1	170.01 (11)	C2—C3—C10—C11	101.14 (14)
C4—O2—C2—C3	−92.79 (14)	C2—C3—C10—C15	−78.33 (17)
C2—O2—C4—C5	−168.40 (12)	O2—C4—C5—C6	179.78 (13)
C2—O2—C4—C9	12.45 (19)	C9—C4—C5—C6	−1.0 (2)
C22—O5—C19—C18	−163.97 (13)	O2—C4—C9—C8	−179.94 (13)
C22—O5—C19—C20	16.4 (2)	C5—C4—C9—C8	1.0 (2)
C3—N1—C1—O1	178.91 (16)	C4—C5—C6—C7	0.8 (2)
C3—N1—C1—C2	−2.36 (11)	C5—C6—C7—Cl1	−179.53 (12)
C16—N1—C1—O1	−5.2 (3)	C5—C6—C7—C8	−0.5 (2)
C16—N1—C1—C2	173.55 (14)	Cl1—C7—C8—C9	179.44 (12)
C1—N1—C3—C2	2.30 (11)	C6—C7—C8—C9	0.4 (3)
C1—N1—C3—C10	−115.47 (12)	C7—C8—C9—C4	−0.6 (2)
C16—N1—C3—C2	−173.81 (13)	C3—C10—C11—C12	−179.70 (13)
C16—N1—C3—C10	68.42 (18)	C15—C10—C11—C12	−0.2 (2)
C1—N1—C16—C17	−172.79 (14)	C3—C10—C15—C14	179.50 (13)
C1—N1—C16—C21	6.7 (2)	C11—C10—C15—C14	0.0 (2)
C3—N1—C16—C17	1.9 (2)	C10—C11—C12—C13	0.3 (2)
C3—N1—C16—C21	−178.63 (13)	C11—C12—C13—N2	−179.27 (14)
O3—N2—C13—C12	−162.24 (17)	C11—C12—C13—C14	−0.2 (2)
O3—N2—C13—C14	18.6 (2)	N2—C13—C14—C15	179.09 (14)
O4—N2—C13—C12	18.5 (2)	C12—C13—C14—C15	0.0 (2)
O4—N2—C13—C14	−160.65 (17)	C13—C14—C15—C10	0.1 (2)
O1—C1—C2—O2	−60.7 (2)	N1—C16—C17—C18	179.53 (12)
O1—C1—C2—C3	−179.12 (17)	C21—C16—C17—C18	0.0 (2)
N1—C1—C2—O2	120.62 (12)	N1—C16—C21—C20	−179.90 (12)
N1—C1—C2—C3	2.21 (10)	C17—C16—C21—C20	−0.4 (2)
O2—C2—C3—N1	−115.16 (12)	C16—C17—C18—C19	0.3 (2)
O2—C2—C3—C10	1.60 (17)	C17—C18—C19—O5	−179.93 (13)
C1—C2—C3—N1	−2.04 (9)	C17—C18—C19—C20	−0.3 (2)
C1—C2—C3—C10	114.72 (12)	O5—C19—C20—C21	179.54 (13)
N1—C3—C10—C11	−159.77 (12)	C18—C19—C20—C21	−0.1 (2)
N1—C3—C10—C15	20.76 (18)	C19—C20—C21—C16	0.4 (2)

### Hydrogen-bond geometry (Å, °)

**Table:**

Cg4 is the centroid of the C16–C21 benzene ring.

**Table:**

*D*—H···*A*	*D*—H	H···*A*	*D*···*A*	*D*—H···*A*
C2—H2A···O4^i^	1.00	2.45	3.287 (2)	141
C3—H3A···O1^ii^	1.00	2.29	3.2439 (17)	159
C15—H15A···N1	0.95	2.58	2.9127 (18)	101
C20—H20A···O1^iii^	0.95	2.50	3.3086 (18)	144
C21—H21A···O1	0.95	2.52	3.1397 (18)	123
C6—H6A···Cg4^iv^	0.95	2.71	3.4145 (17)	131

Symmetry codes: (i) *x*+1/2, −*y*+3/2, *z*−1/2; (ii) *x*−1, *y*, *z*; (iii) −*x*+2, −*y*+1, −*z*+1; (iv) −*x*+3/2, *y*+1/2, −*z*+3/2.

### Table 2 The dihedral angles between the mean planes of the rings in the title molecule (°)

**Table:**

	Ring 2	Ring 3	Ring 4
Ring 1	53.07 (9)	73.19 (9)	6.61 (9)
Ring 2		64.42 (7)	46.85 (7)
Ring 3			79.45 (7)

Ring 1 is the N1/C1–C3 β-lactam ring, ring 2 is the C4–C9 benzene ring, ring 3 is the C10–C15 benzene ring and ring 4 is C16–C21 benzene ring.
